# Yeasts in Liquid Swine Diets: Identification Methods, Growth Temperatures and Gas-Formation Potential

**DOI:** 10.3390/jof6040337

**Published:** 2020-12-04

**Authors:** Birgit Keller, Henrike Kuder, Christian Visscher, Ute Siesenop, Josef Kamphues

**Affiliations:** 1 Institute for Animal Nutrition, University of Veterinary Medicine Hannover, Foundation, 30173 Hannover, Germany; henrike.kuder@uzh.ch (H.K.); christian.visscher@tiho-hannover.de (C.V.); josef.kamphues@tiho-hannover.de (J.K.); 2 Institute for Microbiology, University of Veterinary Medicine Hannover, Foundation, 30173 Hannover, Germany; ute.siesenop@tiho-hannover.de

**Keywords:** yeasts, liquid swine diets, MALDI-TOF, biochemical identification, growth temperature Ancom Gas Production System, *Candida krusei*, *Candida lambica*

## Abstract

Liquid feed is susceptible to microbiological growth. Yeasts are said to cause sudden death in swine due to intestinal gas formation. As not all animals given high yeast content feed fall ill, growth and gas formation potential at body temperature were investigated as possible causally required properties. The best identification method for these environmental yeasts should be tested beforehand. Yeasts derived from liquid diets without (LD − S) and liquid diets with maize silage (LD + S) were examined biochemically (ID32C-test) and with MALDI-TOF with direct smear (DS) and an extraction method (EX). Growth temperature and gas-forming potential were measured. With MALDI-EX, most yeast isolates were identified: *Candida krusei* most often in LD − S, and *C. lambica* most often in LD + S, significantly more than in LD − S. Larger colonies, 58.75% of all yeast isolates, were formed at 25 °C rather than at 37 °C; 17.5% of all isolates did not grow at 37 °C at all. Most *C. krusei* isolates formed high gas amounts within 24 h, whereas none of the *C. lambica*, *C. holmii* and most other isolates did. The gas pressure formed by yeast isolates varied more than tenfold. Only a minority of the yeasts were able to produce gas at temperatures common in the pig gut.

## 1. Introduction

Yeasts, about 600 species of which are known [1], are ubiquitous in nature and can also be found on feedstuffs [2]. They pose a risk factor regarding hygiene in liquid diets associated with off-flavor and loss of nutrients [3,4,5]. Depending on the species or strain, as well as on the growth conditions like temperature, substrate and its aw-value (activity of water), yeasts are able to metabolize numerous sugars, starch, protein, amino acids or even fats, and therefore lead to a loss of nutrients and energy in the feed [3,4,5]. In pig fattening, these energy losses in the feed are particularly undesirable [4,5]. In addition, the flavor and smell of the feed can be negatively affected [6,7]. High cell counts of yeasts in liquid swine diets due to pronounced metabolic activity are often seen in the presence of easily fermentable, low molecular weight sugars [5]. Choosing maize silage for pig feed was used with the aim of feeding the pigs to increase the feeling of satiety without making them fat [8]. The relatively high initial yeast flora of the feed has to be taken into account [8]. Therefore, feed hygiene related to yeast content was of special concern.

In liquid feeds, mostly microflora develops, which is dominated by lactic acid-producing bacteria [7]. A pH-value lower than 5.0, which significantly reduces several bacteria, is often achieved in a shorter time with the use of starter cultures for fermented liquid feeds [3,9,10]. Yeasts are not only able to stay alive but also continue growing in fermented feeds [11], even if the pH-value is 4.5 [12]. 

Besides these complications concerning feed composition and quality, animal health may be affected due to the yeast content in the feed [11,13,14,15]. Hemorrhagic bowel syndrome (HBS), mainly caused by yeasts [13], is supposed to be causally responsible for gastric torsion and gastrointestinal tympani [16], being sometimes associated with liquid feeding [15]. HBS preferentially affects fattened pigs in the second half of the fattening period [16]. Those animals most affected are, as a rule, the better developed pigs in the group [14]. The fact that the affected animals are in excellent health makes this disease of particular economic importance [15].

In feed analyses, yeasts, irrespective of the species, are classified as spoilage indicators in animal feed [17]. A liquid diet with more than 10^6^ cfu yeasts/g original substance (OS) is considered as significantly increased, while less than 10^5^ cfu/g feed OS in liquid feed is considered as normal [18]. On the other hand, selected yeasts are authorized feed additives in human nutrition and animal feedstuffs as they synthesize vitamin B1, B2, B6, B12, folic acid, niacin, pantothenic acid and biotin, as well as containing some minerals (potassium, sodium, calcium, zinc and iron) [1]. For swine diets, viable *S. cerevisiae* is authorized as a feed additive as intestinal flora stabilizers, digestibility enhancers and microorganisms with a minimum concentration of 1 × 10^9^ cfu/kg complete feed (88% DM) [19].

Pathogenicity factors of yeasts have been analyzed to identify high-risk yeasts and their effects on humans and animals. In their study about potential virulence of food-borne yeasts, Rajkowska et al. [20] stated that the ability to grow at 37 °C was crucial; hence, they referred to this as preliminary criterion for pathogenicity. Adaptation to pH-value was also suggested to be a key to pathogenicity, especially important for yeasts entering the digestive tract where the pH-value changes from pH 2 to pH 8 [21,22]. The ability to form biofilms also on abiotic surfaces [21] or even to colonize them is a prerequisite for colonizing the liquid feeding system, which allows the yeasts to stay alive even if the hygiene of the liquid feed was improved [23]. Stalljohann et al. [3] distinguished yeasts according to their ability to produce high or low amounts of CO_2_ with regard to their pathogenicity for swine, but did not mention which species produced the high gas amounts. Such detailed information on these possible indicators of pathogenicity is provided in the present paper.

The hypothesis of this study was that different yeast species could be found in different feedstuffs. For this reason, a comparison of biochemical differentiation and identification with MALDI-TOF was performed to determine the method with the most reliable identification. Presumably, only distinct species would be able to grow and to produce high amounts of gas at 37 °C.

A comparison between gas amounts produced from yeasts measured with the Ancom Gas Production System under defined conditions in a standardized Sabouraud glucose bouillon, had, to the best of the authors’ knowledge, never been carried out previously. This permits a comparison of yeast isolates not only to see whether but also how much gas can be produced by yeasts within a certain time period regardless of feedstuffs. Further studies must clarify whether and to what extent these properties have an influence on the development of diseases such as HBS. These new aspects could then allow to make better predictions concerning the ability of high yeast cell counts in liquid diets to cause clinical problems.

## 2. Materials and Methods

### 2.1. Sample Origin

For our project, samples from farms with liquid feeding common to all samples were collected. We obtained these samples either by contacting farms in the area, or from our own studies, which were also carried out on similar farms. In total, 42 liquid feed samples were analyzed. Of these samples, 33 were submitted for diagnostic purposes to the Institute for Animal Nutrition, University of Veterinary Medicine Hannover, Foundation, Germany. These included common liquid feeds to which no silage had been added, referred to as liquid diets without silage (LD − S). The remaining nine samples obtained from field trials carried out by the Institute for Animal Nutrition, additionally contained whole plant maize-silage (up to 66% DM; liquid diets with silage, LD + S). For collecting the liquid feed samples, a standard laboratory protocol was used for both the submissions and the samples from the studies. The protocol required that the samples were taken fresh, packed directly into a sterile, unbreakable vessel, filled to 2/3 at most, immediately cooled and not sent before the weekend. All samples were processed directly, or in case they arrived late in the afternoon, refrigerated and processed the following morning.

### 2.2. Detection Techniques

Yeasts were isolated and morphologically characterized on Sabouraud glucose agar (SAB-Agar, PO 5096A, Thermo Fisher Scientific GmbH, Bremen, Germany) and then incubated at 30 °C. Only yeasts that grew at the highest decimal dilution levels of the agar plates were considered.

#### 2.2.1. Biochemical Differentiation

Biochemical differentiation of the yeast isolates was performed by ID 32 C strip (bioMérieux SA, Marcy-l‘Ètoile, France). This was performed in accordance with the manufacturer’s instructions. The strip consisted of 32 cavities, each containing a dehydrated carbohydrate substrate, testing the assimilation by the yeast. Pure culture yeast material of 44–48 h-grown subcultures was suspended in 3 mL aqua destillatum. Turbidity was set in accordance with a McFarland standard of 2.0 using a Densitometer DEN-1B (BioSan, Riga, Lettland). From this solution, 250 μL were added to the API C medium included in the test kit. After careful vortexing, 135 μL were transferred from this liquid medium to each well of the test strip. The strip was incubated at 30 °C for 44–48 h. Yeast growth resulted in turbidity of the liquid medium in the cupules, which was visually evaluated. The obtained results were noted on a result sheet. The values corresponding to the positive reactions were then added up within groups. Three results each were added up for a group. Group values were coded into a numerical profile. This was analyzed by means of identification software (APIWEB^TM^, bioMérieux). The results of two of these carbohydrates, *N*-acetylglucosamine (NAG) and lactic acid (LAT), are examined in more detail below. Only results that received good, very good or excellent (classified as “Very good identification”) ratings were evaluated. Rice agar (Thermo Fisher Scientific GmbH), incubated at 25 °C for 44–48 h, was selected for some isolates if the identification software required this deficiency medium, with a cover glass placed over the inoculum for an oxygen-reduced atmosphere. 

#### 2.2.2. MALDI-TOF

In the MALDI-TOF analysis, the sample (e.g., bacteria or yeasts) was ionized by a laser beam. These ions were then accelerated differently depending on their mass and charge. The time required to pass through the length of the flight tube was determined [24]. In this way, a characteristic spectrum can be generated for bacteria or fungi, which usually allows a species diagnosis [24,25]. As an advantage, less time is required for this method compared to biochemical methods [24].

MALDI-TOF analyses were performed on a Microflex LT/SH MALDI-MS Biotyper (Bruker Daltonik GmbH, Bremen, Germany) with the direct smear method (MALDI-DS) and with a formic acid–ethanol extraction (MALDI-EX). The latter is used for hardly soluble bacteria or yeasts.

For MALDI-DS, direct on-plate smearing was performed with yeasts incubated 44–48 h on an SAB-plate at 30 °C. Small amounts of colony material of every isolate were evenly applied with a toothpick to two circles of the target plate (8280800 MSP 96 Target polished steel BC, Bruker Daltonik). After air drying the sample material at room temperature for five to ten minutes, 1 μL of an α-cyano-4-hydroxycinnamic acid (HCCA, 19182, Sigma Aldrich Inc., St. Louis, MO, USA) matrix solution was applied to each circle of the target plate and dried again at room temperature.

For MALDI-EX, a 1 μL loop of 20–24 h-grown yeast material on an SAB-plate was vortexed in 300 μL deionized water at 30 °C; 900 μL of ethanol absolute, HPLC-grade, was added and it was vortexed again. The samples were pelleted by centrifuging for two minutes (13,000 U/min; approximately 3000× *g*); the supernatant was discarded, centrifuged and then the supernatant was discarded again. The pellet in the Eppendorf tube was air dried for three to five minutes and resuspended in 40 μL 70% formic acid. After adding 40 μL acetonitrile (ACN, Acetonitrile HPLC Gradient Grade, 20060.320 VWR International Inc., Radnor, PA, USA), this was followed by the same centrifugation step as above. Eppendorf tubes were taken carefully out of the centrifuge and 1 μL of the supernatant was applied to a circle of the target and air dried for 5 min at room temperature. Immediately afterwards, 1 μL of the same matrix solution that had been previously used for the MALDI-DS method was applied to a circle of the target and air dried for 5 min at room temperature.

For the control of both methods, 1 μL BTS (Bacterial Test-Standard, 8290190 Bruker IVD Bacterial Test-Standard) was placed on every target plate and, additionally, a control strain, *E. coli* DH5 α, was tested on every target plate in two circles.

Each sample was analyzed by a Microflex LT/SH MALDI-TOF MS in the linear mode across a mass-to-charge ratio range between 2000 and 20,000. The obtained data were analyzed automatically by using the MBT Compass Library BDAL and MBT Flex Control software, BTyp2.0-Sec.Library, 1.0. Every strain was tested in two circles, with a decreased concentration in the second circle. The result from the two circles that achieved the higher score value was used for analysis.

The identification cut-off scores were interpreted as per Bruker’s recommendation scores as follows: obtaining scoring thresholds between 2.30 and 3.0 suggested highly probable species identification; 2.00–2.29, probable species identification; 1.70–1.99, identification at the genus level was postulated; whereas cut-off scores <1.70 indicated no reliable identification.

#### 2.2.3. Method Comparison

The results “excellent identification” and “very good identification” adopted in the software APIWEB^TM^ were equated to “highly probable species identification” from the MALDI-TOF analysis. “Good identification” was equated to “probable species identification”; “probable genus identification” was equated to “good identification at genus level”; and “doubtful profile” and “no identification” were equated to “unacceptable profile”.

### 2.3. Temperature Comparison

After cultivating the yeasts from liquid feeds on SAB-agar and subcultivating a single colony, a subculture on two SAB-agar plates was produced for the temperature comparison. One agar plate each was cultivated in an incubator at either 25 °C or 37 °C for 48 h. The colony growth (diameter) was compared visually.

### 2.4. PH-Value

Using a calibrated glass electrode (HI 2211 pH/ORP Meter, Hanna Instruments Inc., Woonsocket, RI, USA), pH-values were measured. Results of 25/33 LD − S and 16/17 LD + S were obtained immediately after dividing the samples for further microbiological testing.

### 2.5. Gas Pressure Measurement

To measure yeast activity in gas production, 40 selected yeast isolates were examined for 24 h at 37 °C with the ANKOM RF Gas Production System (ANCOM Technology, Macedon, NY, USA), which remotes pressure under controlled pressure measurements and records these on a standard Excel spreadsheet. Gas production curves were generated. In 100 mL glass bottles containing 100 mL Sabouraud glucose broth (SAB-B; CM 0147 B, Thermo Fisher Scientific GmbH), a 10 μL yeast suspension, McFarland standard 0.3 (Densitometer DEN-1B, Biosan, Riga, Lettland), in a physiologic salt solution, was added. SAB-B conforms to the parameters from the harmonized EP/USP/JP Microbial Limit Testing for the microbial enumeration tests and tests for specified microorganisms. The bottles were placed on magnetic stirring panels (MIXdrive magnetic e motion with Mixcontrol 20, 2mag AG, Munich, Germany) for permanent mixing at 210 rpm. Gas pressure was measured over a 24-h period, taking into account that feed normally does not normally stay longer in animals’ gastrointestinal tract. Line charts of the cumulated gas production were generated with the Ancom Gas Pressure Monitor. Each isolate was tested at least twice.

The results were divided into two groups. A very small pressure increase (<100 mbar) at the beginning was also observed if no further gas was produced thereafter. If less than 100 mbar of cumulated pressure within 24 h was observed, the result was determined as negative. If a yeast was able to produce more than 800 mbar within 24 h, the result was determined as gas production. This value was defined based on the results found, because no yeast produced gas amounts between 100 and 800 mbar.

### 2.6. Statistics

Data were statistically analyzed using the SAS^®^ Enterprise Guide^®^ (version 7.1, Fa. SAS Institute Inc. Cary, NC, USA). Pearson’s chi-square homogeneity test and Fisher’s exact test, used to analyze qualitative analytical characteristics, were applied to check if a yeast was found significantly more with one feed; if one of the identification tests found significantly more reliable results; whether yeasts grew better at a certain temperature; or if yeasts built up a distinct pressure at 37 °C within 24 h. Fisher’s exact test was used especially for low absolute frequencies.

## 3. Results

In total, 95 morphologically different yeast colonies (color, size and surface structure) were isolated from a total of 42 feed samples. In each feed sample, one to four different yeast species were found.

### 3.1. Identification

In spite of a different morphology, yeast identification led to the same result for 15 yeast isolates. Yeasts diagnosed twice in the same sample were not considered in the evaluation of the number of yeast species or species of yeast-like organisms that were found in the respective feed sample.

The isolates originated from six genera (*Candida* (76.25%; *n* = 61)*, Geotrichum* (7.5%, *n* = 6)*, Trichosporon* (3.75%, *n* = 3)*, Saprochaete* (2.5%, *n* = 2), *Rhodotorula* (1.25%, *n* = 1), *Pichia* (1.25%, *n* = 1) and non-identified yeasts (7.5%, *n* = 6)). A total of 19 different yeast species were identified.

In Table 1, the most often isolated species are listed. Less frequently isolated (one to three times) were *C. pelliculosa* (*n* = 3, 2× LD + S, 1× LD − S), *C. valida* (*n* = 3; 2× LD − S, 1× LD + S), *Sap. suaveolens* (*n* = 2, both from LD − S), *C. rugosa* (*n* = 2, both from LD − S), *C. kefyr* (*n* = 1, LD − S), *C. variabilis* (*n* = 1, LD − S), *C. spherica* (*n* = 1, LD − S), *T. asahii* (*n* = 1, LD + S), *T. coremiiforme* (*n* = 1, LD − S), *T. laibachii* (*n* = 1, LD − S), *P. manshurica* (*n* = 1, LD − S), *Rhodotorula mucilaginosa* (*n* = 1, LD − S) and *Candida* spp. (*n* = 1, LD − S). Six isolates (5× in LD − S; 1× in LD + S) were not reliably identified.

**Figure 1:**
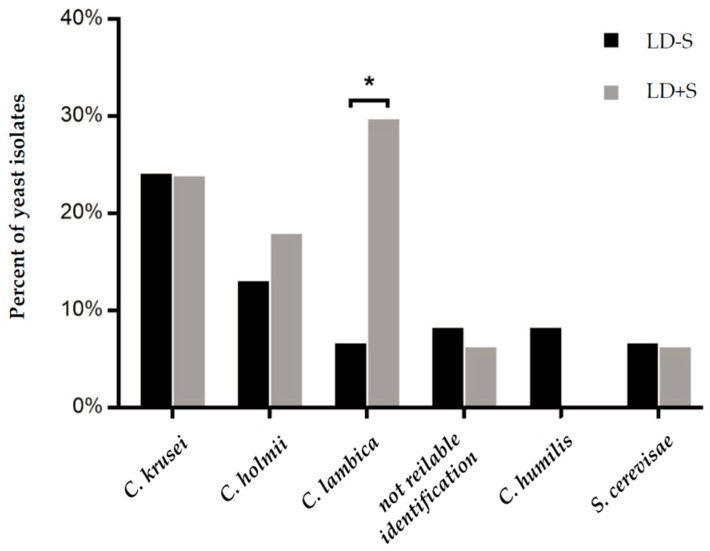
Incidence of yeasts according to their feed origin. An asterisk (*) indicates a significant difference (* *p* < 0.05). *C*. = *Candida*; *S*. = *Saccharomyces*.

Only *C. lambica* was determined to have a significantly higher incidence in LD + S (*p* < 0.0126). The occurrence in LD − S or LD + S did not significantly change (Figure 1).

If, despite different colony morphology, two identical yeast species were diagnosed in one feed sample, only one yeast species was further examined. In 33 LD − S, 7/70 yeast colonies with different morphologies were selected, but identified as the same yeast species within the same diet. In LD + S, 8/25 morphologically different yeasts were identified as the same yeast within the same diet. Therefore, 63 yeasts from LD − S and 17 yeast from LD + S were further examined. As a result of this, significantly more different yeast morphologies were observed from the LD + S samples (*p* < 0.0211).

On the other hand, no differences between the two feeding groups were found regarding the actual (not morphologically) different yeasts. In both diet groups, an average of 1.9 different yeasts per feed sample were diagnosed: 63 different yeasts of 33 LD − S diets, and 17 different yeasts of nine LD + S diets.

#### 3.1.1. Method Comparison

The comparison of methods revealed the most reliable results with MALDI-EX (78.75% reliable species identification), closely followed by ID32C with 75.0% reliable results. These test results did not differ statistically significantly (*p* < 0.6762). Among the reliable results, ID32C provided differentiation of ten isolates (8%), seven isolates with species identification and three isolates were at least identified up to genus level, which MALDI-EX was not able to differentiate. Hence, taking the results of both methods together, 71 (88.75%) of all isolates were identified up to species level. A probable identification or rather identification only at genus level was possible in three (3.75%) isolates. No identification with any of the three diagnostic methods was made for 6/80 (7.5%) yeast isolates. Only few results (37.5%) were provided by MALDI-DS (Table 2). When evaluating the reliable results, both MALDI-EX and ID32C differed significantly (both *p* < 0.001) from the MALDI-DS. No identification with any of the three diagnostic methods was made for 6/80 yeast isolates (7.5%). For better comparison, the results of all three methods were presented together in one table. However, the yeast names (according to their teleomorphic or anamorphic growth) given in the results varied sometimes according to the evaluation software.

Some yeasts were diagnosed more accurately with one or the other method. Five isolates differentiated with MALDI-EX as *C. humilis* were diagnosed as *C. holmii* in ID 32 C. *C. humilis* was not included in the database used to evaluate the ID 32 C. Both of these species had very similar biochemical reactions. Therefore, identification with MALDI-EX was chosen to be more accurate.

The two yeasts identified as *C. pararugosa* in MALDI-EX were identified as *C. rugosa* in ID 32 C. Results in MALDI-EX were only 1.88 and 1.79, respectively. Therefore, they had to be named according to ID 32 C, where the results for both isolates revealed very good identification scores, namely, 99.8% and 99.5% for *C. rugosa*. On the other hand, *C. pararugosa* was not included in the database used to evaluate the ID 32 C.

Even with the formic acid extraction method, the slimy and red growing *Rhodotorula* (*R*.) *mucilaginosa* could not be detected with MALDI-TOF; there was no reliable identification, although it was registered in the database.

**Figure 2:**
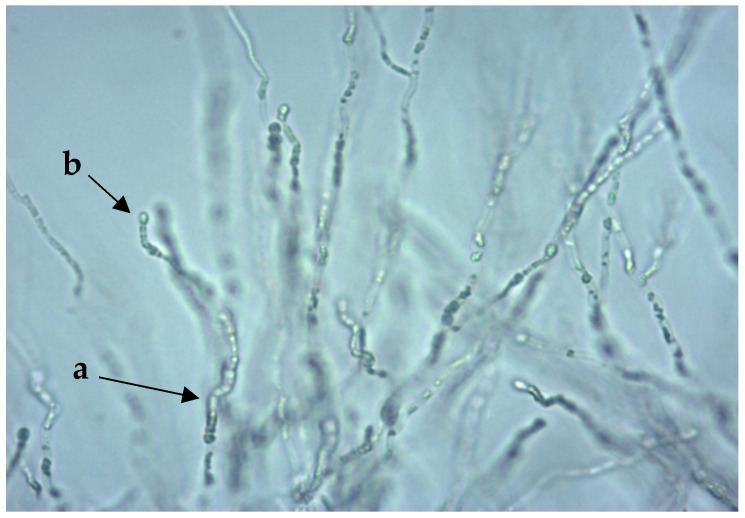
*Trichophyton coremiiforme* on rice agar: arthrospores (**a**) and blastospores (**b**). 400× magnification.

In the ID 32 C, two isolates were diagnosed as *Cryptococcus* (*C. curvatus and C. laurentii*), which showed no mucus capsule in the Indian ink preparation, but showed hyphal growth, arthro- and blastosporogenesis on rice agar under the cover glass (Figure 2), and were identified in MALDI-EX as *T. coremiiforme* and *T. laibachii* (Table 3). On the other hand, two *T. asahii* isolates could be recognized well or very well by both methods.

Six isolates had a score between 1.79 and 1.98 in the MALDI-EX. Of these isolates, *S. cerevisiae* (score 1.98) and *C. holmii* (score 1.97) had the same result in ID32C (see Table 4), with very good identification. Furthermore, two isolates were identified as *C. pararugosa* in the MALDI-EX (with scores 1.88 and 1.63). *C. pararugosa* was not included in the identification software (APIWEB^TM^, bioMérieux). The remaining two isolates consisted of *Saprochaete* (*Sap.*) *suaveolens* (score 1.88), which was diagnosed as a *Geotrichum* spp. in ID32C, and *Pichia occidentalis* (score 1.83), diagnosed with 99.7% as *C. krusei* in ID32C. Bearing in mind that *Sap. suaveolens* was formerly diagnosed as *Geotrichum fragrans*, the diagnosis made by MALDI-EX was most likely the one with the currently correct name. The name of the yeast in ID32C was probably out of date; the yeast was still correctly identified. Only one isolate was differently identified by the two methods as *P. occidentalis* (MALDI-TOF) and *C. krusei* (ID32C; Table 4).

#### 3.1.2. Biochemical Reactions

In total, all the investigated yeasts were able to assimilate glucose and no yeast grew in the cupule where no substrate was present (cupule F). These reactions were considered as the positive growth control or negative control (no contamination). From the large number of biochemical reactions, two of them will be examined in more detail in the following section, since the ability of the yeasts to metabolize them could be an advantage, especially in maize silage.

##### Metabolization of N-Acetylglucosamine (NAG)

In the ID 32 *C*-Test, 27 yeasts from a total of 63 yeasts in the LD − S samples were able to metabolize *N*-acetylglucosamine and 36 yeasts were not. In LD + S, 11 yeasts were able to build *N*-acetylglucosamine and six yeasts were not. Despite the fact that this is insignificant (*p* = 0.0788), the ability to build NAG was more often seen in yeasts from LD + S.

##### Metabolization of Lactic Acid (LAT)

In the ID 32 *C*-Test, 36 yeasts of a total of 63 yeasts in the LD − S samples were able to metabolize lactic acid and 27 yeasts were not. In LD + S, 12 yeasts were able to metabolize lactic acid and five yeasts were not. These results were insignificant (*p* = 0.4075). Nevertheless, the ability to metabolize lactic acid could be found more often with yeasts that had to stay alive or even grow in maize silage than for yeasts in LD − S.

### 3.2. Temperature Comparison

Most yeast isolates (*n* = 47; 58.75%) formed larger colonies at 25 °C than at 37 °C, among them, 14 isolates (17.5%) did not grow at 37 °C at all. These included ten isolates from LD − S (3× *C. holmii,* 1× each for *C. humilis*, *C. lambica*, *T. laibachii*, *C. pelliculosa*, *Geotrichum* spp., *S. cerevisiae* and an isolate not identified) and four from LD + S (3× *C. holmii* and 1× isolate not identified). Among all yeasts, which grew better or only at 25 °C, many isolates of *C. holmii, C. humilis* and *C. lambica* were found. Only 23.75% of isolates grew better at 3 °C compared to 25 °C; this was often the case for *C. krusei* (12/19 isolates) and *S. cerevisiae* (4/5 isolates). Additionally, *C. kefyr* (1/1), *C. holmii* (1/11 isolates) and one isolate that could not be identified (1/6) showed better growth at 37 °C. All *S. cerevisiae* isolates were harvested from LD − S samples. Larger colonies at 37 °C were formed from nine *C. krusei* isolates gained from LD − S and three from LD + S, while three isolates from LD − S and one from LS + S formed larger colonies at 25 °C; one isolate showed equal colony growth at 25 °C or 37 °C. Hence, for *C. krusei*, no difference was observed, regardless of which feed it was isolated from. When comparing both feeds, it was noticeable that especially yeasts isolated from LD + S grew poorly at 37 °C (see Table 5). Better growth at 37 °C than at 25 °C for yeasts harvested from LD + S was only seen for three isolates, all of which were *C. krusei.* However, there was no statistically significant difference (*p* < 0.3862) between the two feed sources concerning growth performance (colony size) of the yeasts at either of the temperatures.

### 3.3. pH-Value in Liquid Swine Diets

The pH-values of LD − S (n 25/33) ranged from 3.87–5.78, while the pH-values of LD + S (*n* = 8/9) achieved higher pH-values ranging from pH 4.79 to pH 5.61. Related to the feed origin, 44 yeasts isolated from LD − S were harvested from liquid feed, with an average pH-value of 4.59. Yeasts gained from LD + S were harvested from liquid feed, with an average pH-value of 5.51. *C. krusei* was isolated from liquid swine diets with the lowest (pH 3.87) and highest pH-values (pH 5.78) as well. *C. humilis and C. holmii* were found in diets with lower pH-values (pH 3.9 to pH 5.11), whereas *C. lambica* was isolated once from a diet with a pH-value of 4.45. However, other isolates were harvested from diets with higher pH-values (ranging from pH 4.97 to pH 5.61).

### 3.4. Gas Production

The results of the duplicate testing of each isolate showed small deviations, possibly caused by small differences in cell counts at the beginning as well as differences in replication time and counts of spores formed by each yeast cell during the 24-h incubation period.

Only two groups were formed: yeasts that produced virtually no gas within 24 h at 37 °C and yeasts that produced more than 800 mbar. A further subdivision of the yeasts into groups producing little or a lot of gas was omitted, because too little information was available from the literature as to which quantities could be classified as a lot or little.

No yeast produced gas amounts between 100 mbar and 800 mbar. More yeasts harvested from LD − S produced gas than yeasts that were found in LD + S, but the quantity was not significant (*p* < 0.2216).

Gas production with more than 800 mbar was observed for a total of 13 (40.6%) isolates (Table 6): 10/11 *C. krusei*-isolates, 2/3 *S. cerevisiae*-isolates, 1/1 *C. kefyr* and 1/1 *C. humilis*. *C. kefyr* formed the highest gas pressure, with 10,419 mbar, followed by the *C. krusei* isolates (7134.5, 7073, 6659, 6487, 6383, 6164.5, 4839, 4147.5, 3954.5 and 3940.5 mbar), both isolates of *S. cerevisiae* (1160.5 and 1466.5 mbar) and *C. humilis* (888 mbar). Eleven of these isolates grew better at 37 °C than at 25 °C within 48 h (see Section 3.2). Nonetheless, two isolates, which also grew better at 37 °C, were not able to produce more than 100 mbar gas in 24 h. These two yeasts were one *C. holmii* and one *C. krusei* isolate harvested from LD + S. The latter one produced these high gas quantities only after a 40 h incubation time. On the other hand, one isolate of *C. krusei*, harvested from LD − S, which grew better at 25 °C than at 37 °C, nevertheless produced 3954.5 mbar gas within 24 h. Only one yeast isolate from LD + S could produce significant quantities of gas under the abovenamed circumstances within 24 h (Table 4).

**Figure 3:**
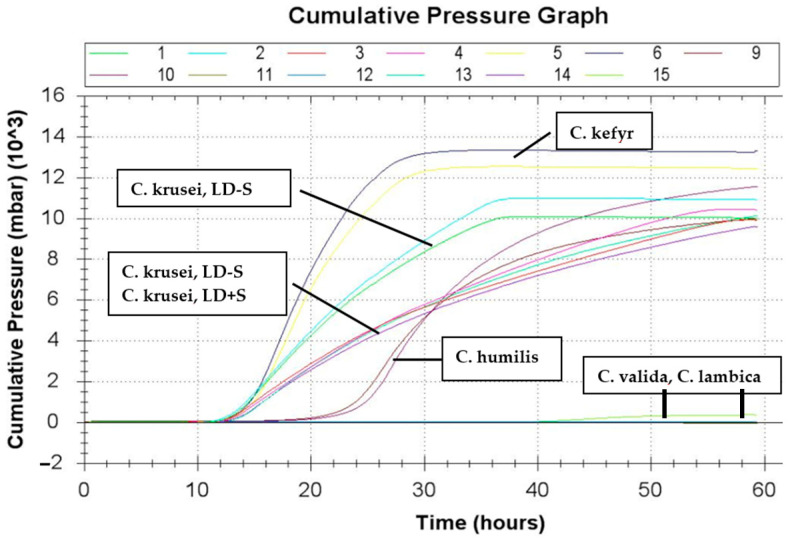
Cumulative pressure graph *C*. = *Candida; C. krusei*, LD − S = isolate of *C. krusei* from LD − S; *C. krusei*, LD-+ = isolate of *C. krusei* from LD + S.

Gas production less than 100 mbar was demonstrated in 5/5 *C. lambica*, 4/4 *C. holmii*, 3/3 *Trichosporon* spp., 3/3 *G. silvicola*, 2/2 *C. pelliculosa*, 2/2 *C. rugosa*, 1/1 *Sap. suaveolens*, 1/1 *Candida* spp., 1/1 *C. valida*, 1/1 *P. manshurica,* 1/1 *C. spherica*, 1/3 *S. cerevisiae* and 1/11 *C. krusei.* Yeasts that showed some signs of growth or grew particularly well at 37 °C within 48 h showed different reactions. Some isolates needed more than 24 h to produce high amounts of gas (Figure 3). Some isolates did not produce amounts greater than 100 mbar, even within a given 60-h period. In Figure 3 such yeasts are *C. valida* and *C. lambica*. Therefore, their curves in Figure 3 are so close to the x-axis they are hardly visable, just like the curve of the control (sterile SAB-bouillon without yeast isolate).

The highest correlation between yeast growth at 25 °C and 37 °C within 48 h (see Section 3.2) and gas formation was found in yeasts that did not grow at 37 °C at all. None of these yeasts (8/8) were able to produce gas during the 24-h incubation period at 37 °C.

## 4. Discussion

Increased numbers of yeasts in liquid feed for pigs has been the subject of some publications in previous years [10,11,12,16,26,27,28]. Some publications compared yeasts in liquid feed for pigs with different feed composition taken from different stables or with and without the addition of starter cultures. However, none have yet compared the yeasts in liquid swine diets with and without maize silage, with the identification results derived from two methods.

### 4.1. Identification

As in most other studies, the genus *Candida* (*C.*) was found most often in our research study. *C. krusei* was found most often in LD − S and in all samples as a whole, whereas *C. lambica* was found most often in LD + S. In addition to these two species, another 17 yeast species from six genera were also diagnosed in this study.

#### 4.1.1. *C. krusei*

Overall, the most often found yeast in this study, *C. krusei*, was isolated from feed samples with the lowest and highest pH-values. From the literature, it is known to grow at low pH-values [29], ferment up to a pH-value of 3.6 [30] and can also form biofilms [31]. These properties are likely to be beneficial for yeast persistence in the liquid feed and the feeding system. In part, *C. krusei* is capable of pseudohyphae formation and mostly of growing at 37 °C, both characteristics that could contribute to HBS.

*C. krusei* is responsible for about 2% of yeast infections caused by *Candida* species in humans [32]. *Pichia kudriavzevii, Issatchenkia orientalis* and *Candida glycerinogenes* are proven to be the same yeast with collinear genomes 99.6% identical in DNA sequence. Under these names, the yeast is used for industrial-scale production of glycerol and succinate, and is also used to make some fermented foods [32]. The latter use in fermented foods also explains the frequent occurrence in liquid feed for pigs, which also has a low pH value (see Section 3.3).

#### 4.1.2. *C. lambica*

The significantly higher presence of *C. lambica* in LD + S is possibly due to maize silage in the feed but could also be due to the lower storage temperature of the maize silage outdoors during winter [33]. As LD + S samples were gained from the institute’s own research projects, it is known that animals did not develop HBS or any other disease and that they ate a lot more with the ad libitum feeding of LD + S in comparison to the previous feed intake with commercial feed (Jörling, 2017) [8]. This was observed, although the yeast content of the feed was temporarily more than 1 × 10^8^ cfu/g feed (Jörling, personal observations, results of which have not yet been published). Olstorpe et al. [26] discovered that *Pichia fermentans* (*C. lambica*) was dominant in all their experiments. They assumed that *C. lambica* was able to improve palatability as it has been described to improve the flavor composition during wine- and cheese-making [26]. Presumably, the yeast species within the diet might be more important than the orientation values, which are the same for all yeasts when the hygiene status of the liquid diets is under debate.

#### 4.1.3. Yeasts from Liquid Diets for Pigs

In the present study, mostly *C. krusei*, *C. holmii*, *C. lambica*, *S. cerevisiae*, *C. humilis* and *Geotrichum* spp. were identified (see Section 3.1, Table 1). Together, they accounted for 66.5% of all yeast isolates. Other species could not be identified (7.5%, Table 1) or were only detected in lower proportions (26%; see Section 3.1).

Middelhoven et al. [33] observed, in whole-crop maize ensiled for two weeks, similar yeast species compared to those found in LD + S. They predominately found *C. holmii, C. lambica, C. milleri* (current name: *C. humilis*), *Hansenula anomala* (current name: *Wickerhamomyces anomalis,* anamorph: *C. pelliculosa*) and *Saccharomyces dairensis* [33], whereby only the latter yeast did not occur in our study. The comparability of the yeast species in both studies could indicate that it is not so much the storage over winter but rather the substrate that influences the yeast occurrence.

The biochemical profiles of both yeasts are very similar and therefore sometimes misidentified [34]. Both yeasts are able to assimilate mostly glucose, lactose, glycerol, inositol and N-acetylglucosamine, while xylose is only metabolized from *C. lambica*. On the other hand, the next frequently identified yeasts, *C. holmii, C. humilis* and *S. cerevisiae*, cannot perform this metabolic function, with the exception of glucose. Instead, they metabolize galactose and raffinose, and, in part, trehalose (*C. holmii and C. humilis*), sucrose (*C. holmii* and partly also *S. cerevisiae*) as well as maltose (*S. cerevisiae*). This could suggest that the assimilative capacities of the yeasts are not essential for their presence or absence in different liquid feeds for pigs.

Likewise, many different yeasts were identified in studies on yeast determination from liquid feed samples for pigs, and different species dominated in the different feed samples [6,26,27,28].

Similarities to the isolated yeasts in the present study were observed in the studies by Olstorpe et al. [26], who examined liquid feeds based on a cereal grain mix and wet wheat distiller’s grain with and without starter cultures. Without starter cultures, they observed *P. fermentans* (*C. lambica*), *C. pararugosa*, *C. rugosa*, *P. galeiformis* (current name: *P. mandshurica*), *T. asahii, Issatchenkia orientalis* (*C. krusei*, *P. kurdriavzevii*), *C. ethanolica* and *C. vini*. These yeasts were found in the present study as well, except the last three mentioned ones.

Olstorpe et al. [26] isolated *C. kefyr* from wheat-based liquid feed as the dominant yeast, which was isolated only once in the present study. This previous publication also found *C. krusei*, *C. pelliculosa* and *Pichia membranaefaciens* (*C. valida*). Plumed-Ferrer and Wright [35] most frequently observed *K. exigua* (*C. humilis*), *Debaromyces hansenii* and *Pichia derserticola* in fresh batches of liquid feed, of which only *K. exigua* was often observed in the present study. On the other hand, in this previous study, other yeasts were also isolated, such as *Pichia kurdriavzevii* (*C. krusei*), *S. exiguous* (*C. holmii*), *Pichia membranaefaciens* (*C. valida*) and *Wickerhamomyces anomalus* (*C. pelliculosa*), which were identified in the present study, too.

Significantly more morphologically different yeasts were observed in LD + S (see Section 3.1), which had lower pH-values than conventional feed and were stored outdoors during winter, which, as a consequence, were exposed to changing temperatures. These different colonial morphologies could be a result of changing environmental conditions, as explained in previous publications [36,37].

The genera *Geotrichum* and *Trichosporon* are classified as yeast or yeast-like organisms, but *Geotrichum* was formerly classified as a mold [38,39,40]. The colony morphology is very similar to other yeasts and therefore was described in many previous studies concerning yeasts in liquid swine diets [25,27,28], so that comparability with other studies is possible. *Saprochaete suaveolens*, formerly classified as *Geotrichum fragrans*, is also classified as a yeast or yeast-like organism (mycobank.org [41]). Hereafter, for the sake of simplicity, all genera are referred to as yeasts, even if the term yeast or yeast-like organism would be more accurate.

#### 4.1.4. Method Comparison

The present study compared different methods for identifying yeasts to find the best method for the chosen substrate and the yeasts contained in it. From previous studies [6,10,26,28,42], it was known that many tests for identifying yeasts from the environment produce fewer results than those from clinical material [43,44,45]. Additionally, different databases on which different test procedures are based also influence the obtained results [45,46].

For clinical samples consisting mainly of *Candida* spp., the method of MALDI-TOF outperformed the diagnosis capacities of the phenotypic tests by reducing the delay in results and improving the reliable identification rate at species level [43]. On the other hand, this method requires significantly higher acquisition costs for the equipment. Therefore, this method was compared with the ID 32 C test, which has virtually no purchase costs.

##### ID32C

In our study, 5.2% less reliable results were observed with the ID 32 C test in comparison to MALDI-EX. Nevertheless, in individual cases, correct identification could only be made with this simple biochemical method (see Table 4). *C. rugosa* was twice identified with more than 99.5% accuracy as “very good identification”, while MALDI-EX identified these two yeast isolates as *C. pararugosa*. The latter was not included in the ID32C-database. Considering the fact that several authors [44,45,47] proposed a lower identification score for the yeast identification with MALDI (see below), perhaps the MALDI results are the correct ones. In the case of *Geotrichum* spp. and *Saprochaete suaveolens*, the situation is similar. *Saprochaete suaveolens* was not included in the database of the ID32C test. Comparable to the finding in the present study, namely that *Rhodotorula* was better identified with ID32C, Olstorpe et al. [6] reported that, with the applied PCR fingerprinting, two *Rhodotorula glutinis* isolates were incorrectly classified as *Cryptococcus satoi* or *Pichia membranaefaciens*, but correctly identified with ID32C.

The ID32C test can be easily performed in any laboratory and does not require an expensive device. In addition to species identification, the biochemical test has the advantage of showing which enzymes can be produced by the respective yeast isolate. This, in turn, could allow or exclude opportunities for identifying which feed components could be metabolized by the yeast.

##### Selected Biochemical Reactions of the ID32C-Test

**Metabolization of *N*-Acetylglucosamine (NAG):** More yeasts from LD + S, even if not significant, were able to metabolize the amino sugar NAG. This is the monomeric constituent of chitin, which is one of the most abundant renewable resources found in nature [48]. The uptake of NAG into the yeast cell, its metabolites in the cell and conversion to cell wall formation have already been described for various yeasts [48]. The cell wall reinforced by NAG (chitin) offers protection against low pH-values in the environment [22]. Although the pH-values in the LD − S were not significantly lower than those of LD + S, the prolonged period of survival in silage (see below) may have led to the ability of yeasts to metabolize NAG.

**Metabolization of Lactic Acid (LAT):** More yeasts from LD + S were able to metabolize lactic acid. As this finding is not statistically significant, the ability to metabolize lactic acid obviously is not a prerequisite for yeasts in LD + S. Lactic acid bacteria are the predominant group of bacteria found in maize silages, and are able to multiply in liquid feed, lactic acid being a main product of their metabolism [49]. Maize silage used for LD + S in the present study was kept outdoors during winter and early spring until it was fed to the animals in late spring and early summer. Being able to use a substrate present in the environment is presumed to be an advantage for yeasts [50,51], which have to survive in these conditions for a long time.

**MALDI-DS:** The less time-consuming and less expensive MALDI-DS reduced the identification rate significantly (*p* < 0.001) by more than half compared to MALDI-EX (37.5% vs. 78.75% reliable identification). Thus, the use of this method is clearly limited, at least if different yeasts are to be identified from environmental samples. In contrast to bacteria, yeasts possess a thick and chitinous cell wall, which might lead to the difficulties encountered with the MALDI-DS method [52].

**MALDI-EX:** In our study, 78.75% of the 80 different yeasts could be identified by MALDI-EX and 75.0% by the ID 32 C. While 11 isolates were not identified at all, six isolates achieved only probable results at the genus level (see Table 4). An incorrect diagnosis was observed only once, mistakenly identifying *P. occidentalis* instead of *C. krusei* (see Section 3.1.1).

A comparison of identification of 96 foodborne yeasts with MALDI-TOF and two conventional tests, of which one was ID 32 C, was made by Pavlovic et al. 2014 [53]. In their study, more yeast isolates could be identified with MALDI-TOF than with the ID32C test, too.

The identification rate of the different methods in the present study was comparable to those of others in which yeasts were isolated from the environment rather than from clinical material [6,54,55,56]. As already shown by these and other authors [1,57], none of the methods were capable of reliably detecting all yeast isolates from the liquid feed. Many authors attribute these differentiation failures to the background of the ID32C, MALDI-TOF and other commercially available tests, as these were developed and established for clinically relevant yeasts in human beings and not for yeasts in animal feed [1,45,57]. None of the available methods can be considered as the golden standard for the differentiation of yeasts from liquid feeds. With respect to the low examination costs, low workload, fast availability of results and available databases, which means the highest rate of correct identification, the different methods exhibit advantages and disadvantages.

Although MALDI-EX was the best method for gaining the most reliable identification results in this study, it has to be considered that results from this method are only as good as the underlying database [46]. Vlek et al. [46] identified 61.5% of their yeasts from human patients using the Bruker Daltonic database (BDAL), but improved their identification rate up to 86.8% by adding their database with the in-house database from the Centraalbureau voor Schimmelcultures (Central Bureau for Fungal Cultures) (BDAL + CBS in-house). This allows the assumption to be made that even more yeasts will be identified with this method in the future, if correspondingly relevant data continue to be added, especially for the non-clinical yeasts found in the surroundings. An improvement in the identification results of 845 environmental yeasts by one third was also described by Augustini et al. [45] after developing a supplementary database.

Besides the databases as reason for missing reliable yeast identification, Augustini et al. [45] stated that identification scores <2.00 are not able to unequivocally affirm that the identification at species level is unreliable. They cited studies that showed identification results under 2.00, but with correct identifications. This observation was underlined in the studies by Tan et al. [44]. Repeating MALDI-TOF attempts in 10.2% of the yeast isolates, which had indicated spectral scores as being unacceptable on the first attempt (scores < 2.00), resulted in acceptable scores (>2.00). Most of these achieved a correct identification on the first attempt [44]. The authors concluded that lowering the identification score from <2.00 to <1.70 could reduce the repetition rate [44]. With a cut-off of <1.70, Lee et al. [50] also improved the identification rate of their 284 pathogenic yeasts from clinical samples compared to the required cut-off value of >2.00 [49]. When comparing the results of two different MALDI-TOF systems (Biotyper from Bruker and ASTA MALDI-TOF MS), Lee at al. [50] found that only 39.5% of the isolates with confirmed identification with molecular sequencing met the cut-off score in both systems. The majority of the isolates (58.6%) ranged between 1.70 and 2.00 when using the Bruker Biotyper and scores > 140 using ASTA MALDI-TOF.

Lee et al. [52] performed a formic acid extraction with a shorter protocol. Most of the yeasts obtained from samples of clinically infected humans were identified correctly, but the method failed to identify the slimy *Cryptococcus* spp. Considering the fact that in our study no *Cryptococcus* spp. were found, possibly this shorter, easier and inexpensive method could have provided as good results as MALDI-EX. On the other hand, different *Cryptococcus* spp. were isolated from liquid swine diets in studies by Olstorpe et al. [6]. Therefore, MALDI-EX seemed to be the best method to reliably identify as many yeasts species as possible.

Extending databases, lowering the identification score for yeasts as well as shorter protocols could improve the ratio of reliable results of environmental yeasts with MALDI-EX in the future, so that the results of this method could be highlighted even more.

Various molecular biological methods described in the literature were not included in this study, although previous authors achieved good results [27]. Gori et al. [27] had difficulties in separating the two most commonly occurring yeasts in their study with 26S rRNA sequencing: *C. humilis* (formerly named *C. milleri*; 58.4%) and *C. holmii* (*Kasachstania exigua*; 17.5%), together accounting for 75.9% of all results (*n* = 766 yeasts). They distinguished the two yeasts biochemically according to their sucrose and raffinose metabolism [27]. In the present study, *C. humilis* and *C. holmii* accounted together for 20% of all results (*n* = 16). In retrospect, it can be assumed that the 26S rRNA method would not have been advantageous in these cases.

### 4.2. Temperature

In the present study, clearly more than half of the yeasts (52.9%) grew better at 25 °C than at 37 °C or did not grow at 37 °C at all (23.5%). Those yeasts that did not grow at 37 °C at all will presumably not grow in the intestines of pigs, where the internal body temperature normally still exceeds 37 °C.

Considering only yeasts isolated from LD + S, there are even more isolates that prefer cooler temperatures (see Table 5). An explanation for these yeasts preferring lower temperatures than yeasts from LD − S could be the chosen time of sampling of LD + S in late spring and early summer in the two projects, when the liquid diets were composed. After harvesting the maize plants and making silage in the fall in the respective projects, this was stored outdoors during winter, where yeasts had to cope with low temperatures. Thus, yeasts may have adapted to these temperatures or died. Storing feed materials or liquid diets at cool temperatures possibly reduces the yeast species, which prefer 37 °C, and as a result have little or no impact on gut health.

The present results could also indicate an adaptation of the yeasts to their feed origin and storage temperature. These results were obtained directly after the cultivation of the yeasts from the respective feed (see Section 2.3). Therefore, yeasts had little or no opportunity to adapt to the new temperatures. This is in the broadest sense comparable with the climatic conditions during the long period between the fall and spring. On the other hand, the possibility to adapt would exist at warmer outside temperatures and in case of the pre-fermentation of the liquid feed (24 h, 38 °C), as is sometimes practiced, especially with controlled fermentation [58]. Suutari et al. [59] reported morphological changes in some yeasts that had to adapt to cooler or very warm temperatures in a bouillon. The investigations in this previous study on growth performance at different temperatures was made on agar plates. The possible easier adaptation to new temperature conditions in a bouillon could also be an explanation for the observations that some yeasts only produce gas at 37 °C after a longer period of time (see Section 3.4, *C. humilis* in Figure 3).

Margesin et al. [60] isolated yeasts and bacteria from cold-adapted habitats and classified 60% of the yeasts but only 8% of bacteria to be true psychrophils, which showed no growth above 20 °C, indicating that the remaining microorganisms are able to adapt to warmer temperatures. Yeasts that do not grow or grow very poorly at 37 °C are thought to have little or no effect on gut health [20]. As a result, the lack of or partly low clinical symptoms on farms with a high yeast load in the feed are explicable. Correspondingly, yeasts that did not grow at all or worse at 37 °C than at 30 °C were also found on yeasts obtained from swab samples from milking machines [55]. A large majority of them could not be recovered from the milk collected with these milking machines.

In both groups (LD − S and LD + S), 1.9 different yeasts were identified. On the other hand, significantly more different colonial morphologies of the yeasts were found in LD + S, possibly indicating that temperature could have an influence on morphology, as was also observed by Nadeem et al. [37].

### 4.3. pH-Value

In the present study, the LD + S had on average slightly higher pH-values than LD − S and they contained significantly more *C. lambica.* Whether this connection is accidental or related to the higher pH-value can only be suspected due to the small number of farms of origin. Lack of growth at 37 °C [34], a good smell/taste [26] but no described ability of biofilm formation, as found by the Olostorpe et al. [26], could mean that this yeast is expected to be less harmful as a feed contaminant and for gut health than other yeasts. On the other hand, some yeasts are known to adapt to pH-values, to temperature and to different media [37], so that the safety of *C. lambica* in liquid swine diets still needs to be tested.

For fungi as well as bacteria, one of the most important environmental conditions is ambient pH. Changes in external pH result in phenotypic, metabolic and physical changes of the microorganisms [22]. The low pH-values in liquid feeds, especially fermented ones or those containing silage compared to normal feed for pigs, in general favor yeasts. This is due to the fact that at pH-values < 5.0, many bacteria are not able to stay alive or to grow as fast as they do at higher pH-values [61]. Molds depend on oxygen, but yeasts are able to grow at low pH-values with and without oxygen [3]. Some yeasts are known to be able to adapt to low pH-values in their surroundings by forming a thicker cell wall with chitin (*N*-acetyl glucuronidase) [22]: the high buffering capacity in the cytosol, high H^+^-ATP-ase and/or high endogenous energy reserves of *C. krusei* [29]. Therefore, fermented liquid feeds, especially after controlled fermentation, always poses a certain risk of increased yeast content.

### 4.4. Gas Production

Quantitatively comparing gas production of different yeasts under standardized conditions with Ancom RF Gas Production System was, to the best of our knowledge, performed for the first time. Investigations in a bouillon, produced in accordance with European and US Pharmacopoeia guidelines, allows for a comparison of gas-producing yeasts irrespective of feed or water. The SAB-bouillon provides ideal conditions for yeasts and contains high amounts of glucose (20 g/L). However, the total gas quantities measured do not describe quantities that would also be produced in the feed or in the animal, since the competing flora is always different, and feed is not composed like a bouillon or an agar for yeasts.

Different generation times, sizes and numbers of buds make it difficult to precisely calculate the yeast quantity with density determination or even with quantitative cell counting. Hence, the amounts of gas production were not precisely determined but categorized to two major groups, as described above. Additionally, not the exact yeast numbers per milliliter were determined but only the density by means of the McFarland standard. Exemplarily, for some samples with the density of McFarland 0.3, the yeasts were counted, resulting in 1–4 × 10^5^ cfu yeasts per mL. Thus, these yeast counts are just about acceptable regarding the requirements in liquid feed according to Kamphues et al. [17].

In the present study, slightly increasing gas pressures were also measured for yeasts that did not produce gas at the beginning of the experiment. This could be explained by the rising room temperature during processing to the 37 °C in the incubator.

The gas formation capacity of the yeasts differed very clearly between 888 mbar and 10,419 mbar. *C. kefyr* formed over ten times more gas than one of the *S. cerevisiae* isolates. Only one yeast isolate from LD + S was able to produce higher amounts of gas at 37 °C within 24 h. This was partly caused by its preference for cooler temperatures, as described above. The reason for the differing amounts of gas production of yeasts may to some extent be seen in the lack of oxygen produced in the Ancom Gas Production System, which is also found in the pig’s colon. Some yeasts like *C. sphaerica, C. variabilis, C. kefyr, C. lambica, C. krusei, S. cerevisiae* and *C. pelliculosa* are known to metabolize glucose under anaerobic conditions; variable metabolization is expected from other yeasts like *C. valida, G. candidum* and *G. capitatum*, while *C. rugosa* and *Rhodotorula* spp. are mostly not capable of fermentation [61]. The latter cannot be expected to produce gas amounts under the conditions available in the present study as well as those found in the gastrointestinal tract of pigs. As such, they cannot be expected to cause a disease such as HBS. Comparing the growth of a yeast from liquid feed for pigs at 37 °C and 25 °C can give a good indication of whether a yeast is likely to cause HBS. However, it is not possible to make an accurate prediction because yeasts are partially capable of adapting to temperatures and some yeasts hardly ferment under anaerobic conditions despite growth at 37 °C. On the other hand, no high gas production within 24 h was observed in the present study when a yeast isolate did not grow at 37 °C. Presumably, those yeasts are not supposed to cause HBS. A test of growth at 37 °C would be easy to perform in every laboratory and could give a hint at whether a yeast would be able to grow in a pig’s alimentary tract. Further studies will be needed to clarify which amount of gas production can generally be called high or low. Apart from this, it has to be considered that a yeast, even if it is not able to form a biofilm itself, may colonize the biofilm of the lines of the feeding system. Such yeasts could potentially be capable of adapting to warmer temperatures, especially in the summer months.

### 4.5. Summary

In several studies of liquid feed, samples for pigs’ yeasts were identified, which were also found in the present study. The most commonly detected yeast in our study was *C. krusei*. This is the first study of liquid feed with and without maize silage. In liquid feed with maize silage (LD + S), significantly more *C. lambica* was found.

MALDI-EX provided the most reliable results (78.75%), but the ID 32 *C*-test, easy to perform in every laboratory, was sufficient for confirming 75.0% of the identified yeasts. Both tests together identified 88.75% of the yeasts because some yeasts were only reliably identified with one or the other test. The quicker MALDI-DS-method provided only 37.5% reliable results, this being significantly less than the other two methods. Thus, a formic acid/acetonitrile extraction (MALDI-EX) before analysis should be preferred.

Clearly more than half of all yeast isolates grew better at 25 °C than at 37 °C. Fourteen isolates showed no growth at all at 37 °C. Gas amounts produced by the different yeast isolates differed more than tenfold within a 24-h incubation period at 37 °C in SAB-bouillon measured with the Ancom Gas Production System. Most of the tested *C. krusei* and *S. cerevisiae* but none of the tested *C. holmii*, *Trichosporon* spp., *G. silvicola* and *C. pelliculosa* were able to produce gas. While only one yeast from LD + S was able to produce gas within 24 h, more yeasts (40.6%) from LD − S were able to do so. None of the yeasts that did not grow on the SAB-agar at 37 °C were able to produce high amounts of gas within a 24-h incubation period at 37 °C in the bouillon, presuming that those yeasts could only slightly affect the animals’ health.

Due to the fact that the majority of *C. krusei* isolates were able to grow at 37 °C, produce high amounts of gas, grow in low pH conditions and form biofilms, as is known from the literature, this yeast species seems to be predestinated to grow in liquid diets and to remain in a biofilm in the pipelines serving the liquid diet. Therefore, special interest should be given to this yeast species. The evaluation of yeast levels in liquid feed for pigs has so far only been determined on the basis of the number of yeasts per gram feed. Laboratory values alone could possibly incorrectly estimate the influence of yeasts on the health of the animals as either being too low or too high. Additional investigations are needed to further characterize the effect of each yeast species on pig health. Moreover, investigating the effect of having the storage temperature of the feed significantly below body temperature could be interesting.

## Figures and Tables

**Table 1:** Often isolated yeast species.

Liquid Diet	Often Isolated Yeast Species in %
LD – S (*n* = 63)	*C. krusei* (23.8), *C. holmii* (12.7), *C. humilis* (7.9), isolates not identified (7.9), *C. lambica* (6.3), *S. cerevisiae* (6.3), *G. silvicola* (6.3)
LD + S (*n* = 17)	*C. lambica* (29.4), *C. krusei* (23.5), *C. holmii* (17.6), *C. pelliculosa* (11.8), *S. cerevisiae* (5.9), *C. valida* (5.9), isolates not identified (5.9)
Total (*n* = 80)	*C. krusei* (23.75), *C. holmii* (13.75), *C. lambica* (11.25), isolates not identified (7.5), *C. humilis* (6.25), *S. cerevisiae* (6.25), *G. silvicola* (6.25)

*C.* = *Candida*; *S.* = *Saccharomyces*; *G.* = *Geotrichum*.

**Table 2:** Comparison of performance of methods with 80 yeast isolates in %.

Evaluation Score of MALDI-TOF	MALDI-EX		MALDI-DS		ID-32C	
Highly probable species identification (>2.3)	38.75 (*n* = 31)	78.75 *^,a^ (*n* = 63)	5.0 (*n* = 4)	37.5 *^,b^ (*n* = 30)	47.5 (*n* = 38)	75.0 **^,a^ (*n* = 60)
Probable species identification (2.0–2.29)	40.0 (*n* = 32)		32.5 (*n* = 26)		27.5 (*n* = 22)	
Probable genus identification (1.7–1.99)	7.5 (*n* = 6)	21.25 *** (*n* = 17)	33.75 (*n* = 27)	62.5 *** (*n* = 70)	3.75 (*n* = 3)	25.0 *** (*n* = 20)
No identification (<1.7)	13.75 (*n* = 11)		28.75 (*n* = 23)		21.25 (*n* = 17)	

^a,b^ Different superscripts differ significantly in a row. * Reliable identification: Highly probable species identification and probable species identification; ** Reliable identification: Good and very good identification together; ***: No species identification.

**Table 3:** Isolates differentially diagnosed with MALDI-EX and ID 32C.

Number of Isolates	MALDI-EX	ID 32C
5	***Candida humilis***	*Candida holmii*
2	*Candida pararugosa*	***Candida rugosa***
1	*No identification*	***Rhodotorula mucilaginosa***
1	***Trichosporon coremiiforme***	*Cryptococcus curvatus*
1	***Trichosporon laibachii***	*Cryptococcus laurentii*

Yeasts highlighted in bold represent the selected diagnoses.

**Table 4:** Isolates in MALDI-EX rated as probable genus identification (1.70 and 1.99).

MALDI-EX		ID32C		
Diagnosis	Score	Diagnosis	Identification	%
*C. pararugosa*	1.88	***C. rugosa***	very good	99.8
*Sap. suaveolens*	1.88	***Geotrichum spp.***	very good	99.7
*P. occidentalis*	1.83	***C. krusei***	very good	99.7
*C. holmii*	1.97	***C. holmii***	very good	99.2
*C. pararugosa*	1.79	***C. rugosa***	very good	99.5
*S. cerevisiae*	1.98	***S. cerevisiae***	very good	99.7

Yeasts in bold represent the selected diagnoses *C*. = *Candida*; *Sap*. *= Saprochaete*; *P*. = *Pichia*; *S*. = *Saccharomyces*.

**Table 5:** Growth performance of the yeasts at different temperatures depending on feed.

Yeast Isolates	No Growth at 37 °C	Better Growth at 25 °C	25 °C = 37 °C	Better Growth at 37 °C
LD − S (*n* = 63)	10 (15.9%)	24 (38.1%)	13 (20.6%)	16 (25.4%)
LD + S (*n* = 17)	4 (23.5%)	9 (52.9%)	1 (5.9%)	3 (17.6%)
Total (*n* = 80)	14 (17.5%)	33 (41.25%)	14 (17.5%)	19 (23.75%)

**Table 6:** Gas production (mbar) at 37 °C within 24 h.

Sample	Number	<100 mbar	>800 mbar
LD − S	*n* = 32	19 (59.4%)	13 (40.6%)
LD + S	*n* = 8	7 (87.5%)	1 (12.5%)
total	*n* = 40	26 (65.0%)	14 (35.0%)
